# Crystal structure of IspF from *Bacillus subtilis* and absence of protein complex assembly amongst IspD/IspE/IspF enzymes in the MEP pathway

**DOI:** 10.1042/BSR20171370

**Published:** 2018-02-21

**Authors:** Zhongchuan Liu, Yun Jin, Weifeng Liu, Yong Tao, Ganggang Wang

**Affiliations:** 1 Key Laboratory of Environmental and Applied Microbiology, Chengdu Institute of Biology, Chinese Academy of Sciences, Chengdu 610041, China; 2 Key Laboratory of Environmental Microbiology of Sichuan Province, Chengdu 610041, China; 3 Chinese Academy of Sciences Key Laboratory of Microbial Physiological and Metabolic Engineering, Institute of Microbiology, Chinese Academy of Sciences, Beijing 100101, China; 4 University of Chinese Academy of Sciences, Beijing 100049, China

**Keywords:** crystal structure, 2-C-methyl-D-erythritol 2, 4-cyclodiphosphate synthase, isoprenoid biosynthesis, MEP pathway

## Abstract

2-C-Methyl-d-erythritol 2,4-cyclodiphosphate synthase (IspF) is a key enzyme in the 2-C-Methyl-d-erythritol-4-phosphate (MEP) pathway of isoprenoid biosynthesis. This enzyme catalyzes the 4-diphosphocytidyl-2-C-methyl-d-erythritol 2-phosphate (CDPME2P) to 2-C-methyl-d-erythritol 2,4-cyclodiphosphate (MEcDP) with concomitant release of cytidine 5′-diphospate (CMP). *Bacillus subtilis* is a potential host cell for the production of isoprenoids, but few studies are performed on the key enzymes of MEP pathway in *B. subtilis*. In this work, the high-resolution crystal structures of IspF in native and complex with CMP from *B. subtilis* have been determined. Structural comparisons indicate that there is a looser packing of the subunits of IspF in *B. subtilis*, whereas the solvent accessible surface of its active pockets is smaller than that in *Escherichia coli.* Meanwhile, the protein–protein associations of 2-C-Methyl-d-erythritol-4-phosphatecytidyltransferase (IspD), CDPME kinase (IspE) and IspF from *B. subtilis* and *E. coli*, which catalyze three consecutive steps in the MEP pathway, are analyzed by native gel shift and size exclusion chromatography methods. The data here show that protein complex assembly is not detectable. These results will be useful for isoprenoid biosynthesis by metabolic engineering.

## Introduction

Isoprenoid compounds are the most diverse group of natural products with a broad range of biological functions [1]. Two five-carbon compounds, isopentenyldiphosphate (IPP) and dimethylallyldiphosphate (DMAPP), are the universal precursors for isoprenoid molecules. IPP and DMAPP are synthesized by two different biosynthetic pathways, the mevalonate (MVA) pathway and the 2-C-Methyl-d-erythritol-4-phosphate (MEP) pathway. The MVA pathway is dominant in the mammals, plants, and fungi [2,3], whereas the MEP pathway is discovered in many bacteria, algae, cyanobacteria, eubacteria, apicomplexan parasites, and plant chloroplasts [4–7]. Since the MEP enzymes are both highly conserved and absent from humans, all enzymes in MEP pathway have gained interests as potential targets for antimicrobial drug development [8–11]. In addition to the therapeutic value of the enzymes in MEP pathway, the isoprenoid production through MEP pathway has also been widely exploited, because it has several potential advantages compared with the MVA pathway, including a theoretically better stoichiometric yield and lower oxygen consumption during fermentation [12].

**Figure 1:**
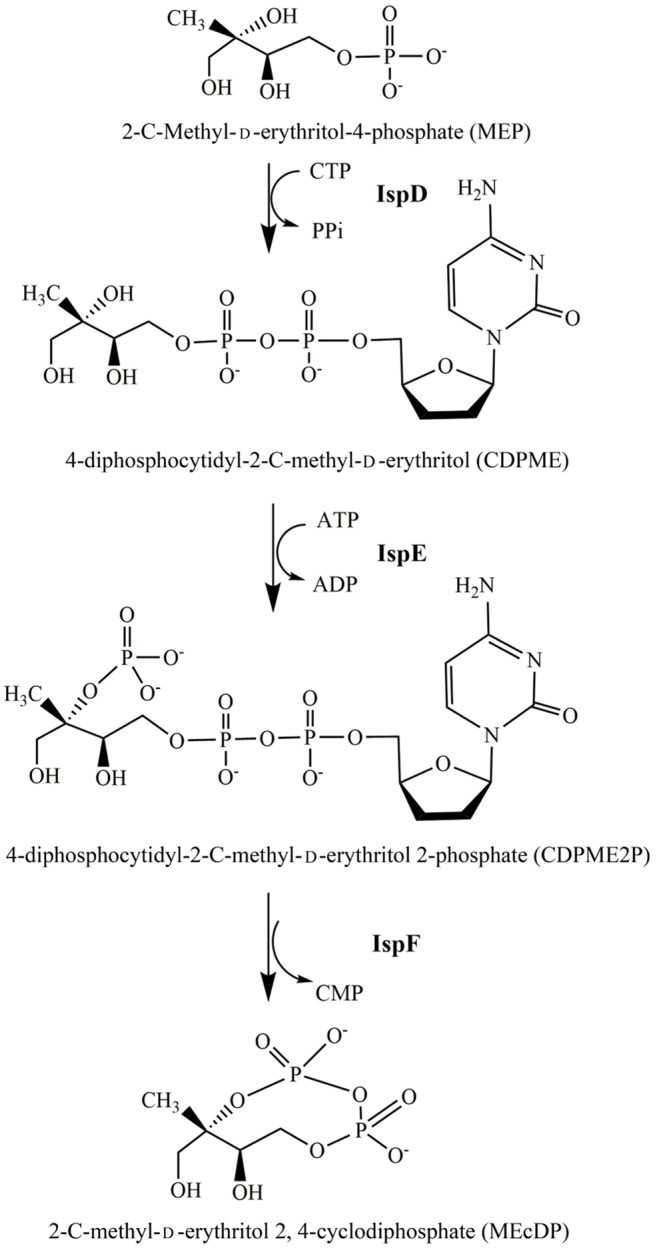
The reaction catalyzed by IspD, IspE, and IspF enzymes in the MEP pathway

The MEP pathway is made of seven consecutive catalytic steps. Amongst them, the enzymes which are encoded by *ispD, ispE*, and *ispF* genes, catalyze the third, fourth, and fifth steps to convert MEP into 2-C-methyl-d-erythritol 2,4-cyclodiphosphate (MEcDP). 2-C-Methyl-d-erythritol-4-phosphatecytidyltransferase (IspD) condenses MEP with CTP to give 4-diphosphocytidyl-2-C-methyl-d-erythritol (CDPME) and PPi [13]. CDPME kinase (IspE) catalyzes the phosphorylation of CDPME to 4-diphosphocytidyl-2-C-methyl-d-erythritol 2-phosphate (CDPME2P) [14]. Finally, the CDPME2P is converted into MEcDP with concomitant release of CMP by MEcDP synthase (IspF) [15] (Figure 1).

Generally, the three genes, *ispD, ispE*, and *ispF* encode monofunctional enzymes in most MEP-dependent organisms. However, bioinformatics analysis indicated that *ispD* and *ispF* genes were fused together in several bacterial species, such as *Campylobacter jejuni, Sinorhizobium meliloti, Brucella melitensis, Helicobacter pylori*, and *Mesorhizobium loti* etc. [16–18]. Biochemical analysis confirmed that the IspDF protein from *C. jejuni* was a bifunctional enzyme catalyzing the third and fifth step reactions in MEP pathway [17]. Furthermore, the crystal structure of IspDF from the *C. jejuni* revealed that the protein presented two distinct domains corresponding to the cytidyltransferase IspD and synthase IspF, respectively [17,19]. The structural arrangement of IspDF was uncommon, because it catalyzed non-consecutive steps in the MEP pathway. Sedimentation velocity studies indicated that possible associations could be formed between bifunctional IspDF and monofunctional IspE from *C. jejuni* and *Agrobacterium tumefaciens* [19]. In addition, by the analytical ultracentrifugation analysis, a complex of ~430 kDa was observed when the individual enzymes IspD, IspE, and IspF from *E. coli* were mixed. This likely corresponded to an assembly of three IspD dimers, three IspE dimers, and two IspF trimers, which could provide 18 catalytic centers and might lead to enhanced metabolic flux in the MEP pathway [19]. However, further research rejected this hypothesis and indicated that there was no evidence for enhancing catalytic rates on the mixture of fused IspDF and individual IspE enzymes from *A. tumefaciens* or any proof of substrate channeling in the mixture of individual IspD, IspE, and IspF proteins [20]. It is controversial whether a multienzyme complex can be formed between IspDF and IspE or amongst the monofunctional enzymes IspD, IspE, and IspF. Further investigations are required to clarify the associations amongst IspD, IspE, and IspF.

Nowadays, the increasing needs for isoprene products prompt the efforts to develop a bio-based process for isoprene production by engineered microorganisms, since it is efficient and environment friendly [21]. Recent studies showed that overexpression of both MEP and MVA pathways in *E. coli* could improve the isoprene yield ~20-fold and 3-fold, respectively, compared with overexpression of the each pathway alone, which demonstrated that the synergy of the MEP pathway and the MVA pathway could be used to improve the production of isoprene in microorganisms [22–24]. *Bacillus subtilis* is a model organism and of significant industrial importance. It was reported that the *B. subtilis* has potential as an excellent isoprene producer [25–27]. However, only few studies on the key enzymes of MEP in *B. subtilis* had been performed [27–29]. In this study, the high resolution structures of IspF and IspF/CMP complex from *B. subtilis* have been determined. Moreover, the interaction of individual enzymes IspD, IspE, and IspF from *B. subtilis* and *E. coli* are investigated. These results will show useful hints on isoprenoid biosynthesis by metabolic engineering.

## Materials and methods

### Materials

Restriction enzymes used in the present study were purchased from New England Biolabs. T4 ligase was purchased from TransGen Biotech. Column resins used for protein purification were purchased from GE Healthcare (U.S.A.). Crystallization screening kits were purchased from Hampton Research. All other chemicals used for preparing buffers and solutions were reagent grade and purchased from Merck, Sigma–Aldrich, and local suppliers.

### Expression and purification of proteins

The *ispD* and *ispF* genes of *B. subtilis* 168 (DSM 23778, DSMZ, Germany) were amplified by PCR from genomic DNA with the 5′/3′ specific primers, which introduced BamHI site and XhoI sites, respectively. The ~699 and ~477 bp amplification products were digested and cloned into pGEX-6P-1; the gene sequence was confirmed by DNA sequencing. The recombinant plasmids were transformed into competent *E. coli* DH5α. Bacterial cells were grown in LB broth supplemented with 100 μg/ml ampicillin. The culture was incubated at 37°C with vigorous shaking. At an optical density (600 nm) of 0.5–0.6, IPTG was added to a final concentration of 0.3 mM, and the culture was further incubated at 16°C for 14–16 h. The cells were harvested by centrifugation; the cell pellets were re-suspended in lysis buffer (25 mM Tris/HCl pH 8.0, 50 mM NaCl, 1 mM DTT) and sonicated on ice. The crude fluid was centrifuged at 11800×***g*** at 4°C for 40 min to remove cellular debris. The proteins were purified from the supernatant by GST Glutathione Sepharose™ 4 Fast Flow column (GE Healthcare), and the GST tag was removed by Prescission Protease (PPase) at 4°C overnight. The eluted IspD and IspF enzymes were further purified by the combination of the Resource Q (GE Healthcare) anion-exchange column and Superdex 75 (GE Healthcare) size-exclusion column. The protein fractions were pooled and determined to be >95% pure by SDS/PAGE. The protein concentration was determined by Bradford method using BSA as the standard. The purified protein was aliquoted for storage at −80°C.

The *ispE* gene of *B. subtilis* 168 (DSM 23778, DSMZ, Germany) was also cloned into the vector of pGEX-6P-1 expression vector. The protein purification process was similar to IspF of *B. subtilis* above. However, the recombinant proteins without GST tag were insoluble; the fusion proteins were prepared by GSH elution from affinity column directly and purified by subsequent purification steps. If no special instructions, the fusion proteins were named as *Bs*IspE.

To exclude the influence of the tag in the association studies, we also purified the recombinant proteins IspD, IspE, and IspF from *E. coli*. The *ispD, ispE*, and *ispF* genes of *E. coli* were PCR amplified from *E. coli* K12 and inserted into the pET-22b expression vector. C-terminal His_6_-tagged proteins were expressed in *E. coli* BL21 (DE3) cells. Tagged proteins were purified using an Ni^2+^-NTA (GE Healthcare) column. The histidine tag was removed by thrombin enzyme. The eluted proteins were further purified by the combination of the Resource Q (GE Healthcare) anion-exchange column and Superdex 75 (GE Healthcare) size-exclusion column. Finally, the purified proteins were essentially homogeneous and >95% pure, as analyzed by SDS/PAGE.

### Protein crystallization and data collection

Crystals of the native IspF protein of *B. subtilis* (*Bs*IspF) proteins were obtained at 18°C by the hanging drop vapor-diffusion method, 1 μl of 10 mg/ml protein in the buffer of 25 mM Tris/HCl pH 8.0, 0.1 M NaCl was added in the reservoir solution containing 0.1 M magnesium formate dehydrate and 12–15% w/v PEG 3350. Crystals of *Bs*IspF/CMP complex were incubated with 5 mM CMP on the ice for 2 h before crystallization and obtained as described above. Crystals were grown over a period of 5–7 days and then dehydrated to the reservoir solution plus 15% of glycerol overnight. Then the crystals were picked from the solution using nylon loops and frozen in liquid nitrogen. X-ray diffraction data of *Bs*IspF crystals were collected at 100 K using synchrotron radiation at Shanghai Synchrotron Radiation Facilities (SSRF) [30]. Datasets were processed and scaled by HKL2000 [31]. Details are shown in Table 1.

**Table 1:** Data collection and refinement statistics

	Native	CMP complex
**Data collection**		
Wavelength (Å)	0.97915	0.97915
Space group	*P*2_1_	*P*2_1_
Unit-cell parameters		
* a, b, c* (Å)	56.96, 89.30, 84.71	57.06, 89.46, 87.99
*β* (°)	100.85	103.80
Monomers per asymemetric unit	6	6
Resolution (Å)	29.05–1.99 (2.06–1.99)^1^	29.21–1.99 (2.06–1.99)^1^
Number of unique reflections	56504 (5662)^1^	57544 (5799) ^1^
CC_1/2_	0.994 (0.877)	0.995 (0.775)
Redundancy	3.7 (3.7)^1^	3.7 (3.8)^1^
Completeness (%)	99.3 (99.9)^1^	98.2 (99.8)^1^
Mean I/σ(I)	15.0 (3.7) ^1^	14.3 (4.8)^1^
*R*_merge_ (%)	8.2 (41.5) ^1^	8.4 (37.4)^1^
Wilson B factor *(Å^2^)*	27.6	26.8
		
**Refinement**		
Reflections (working/test)	53728/2755	54704/2819
*R*_work_/*R*_free_ (%)	18.1/23.1	17.4/21.9
		
*Number of residues*		
Protein	924	932
CMP/Mg^2+^	0/6	4/6
Waters	250	256
		
*Average B factor (Å^2^)*		
Main chain/side chain	35.2/42.7	32.4/40.6
CMP/Mg^2+^	–/32.4	41.6/21.9
Waters	39.7	40.8
		
*Ramachandran plot (%)*		
Favored	97.2	97.7
Allowed	2.8	2.3
Clash score	1.76	1.66
Overall score	1.26	0.98
		
*r.m.s.d.*		
Bond lengths (Å)	0.013	0.013
Bond angles (°)	1.570	1.652

1 The values in parenthesis means those for the highest resolution shell. Abbreviation: r.m.s.d., root mean square deviation.

### Structure determination and refinement

The structures of *Bs*IspF were elucidated by molecular replacement using PHASER [32] from the CCP4 program suite [33]. The starting model was the monomer IspF enzyme from *Shewanella oneidensis* (PDB: 1T0A), which shared 62% sequence identity with the *Bs*IspF and served as a good model for the structure solution. Only one solution was evident. Refinement was performed using the maximum likelihood functions implemented in REFMAC5 [34], while model building and improvement were achieved with COOT [35]. Solvent molecules, metal ions and ligands were positioned after a few cycles of refinement. Non-crystallographic symmetry (NCS) restraints were imposed in the early stages of the analyses and then released as the refinements progressed. Isotropic refinement of the atomic displacement parameters was performed for all atoms. The stereochemistry was checked with the program MolProbity [36]. Details of the overall refinement and final quality of the models are shown in Table 1. The program *PyMOL* (http://www.pymol.sourceforge.net/) was used to prepare structural figures.

### Native gel shift

According to different shift distance of different components, the native gel shift could show whether a stable complex could be formed by different individual proteins. The control groups were only one component protein and corresponding buffer. The experimental groups were carried out using two or three proteins amongst IspD, IspE, and IspF (the concentration of these proteins were 2.5 μg/μl, respectively), 10% glycerol, 25 mM Tris/HCl pH 8.0, 100 mM NaCl in final volume 12 μl; meanwhile, adding 1 mM MgCl_2_, 1 mM MEP, and 1 mM CTP if the IspD was present in the reaction system. The reaction was incubated at 30°C for 30 min. Finally, the samples were analyzed by a 8% native page in 1× TBE buffer for 2.5 h at 200 V at 4°C. The gel was photographed by an imaging system.

### Size exclusion chromatography

Size exclusion chromatography is also applied to investigate whether a stable complex could be formed by different individual proteins. The interactions amongst the IspD, IspE, and IspF were studied using an AKTA purifier and a 24 ml (1 × 30 cm) Superdex75 10/300 GL or Superdex200 10/300 GL size exclusion chromatography column. Typically, size exclusion chromatography was carried out at buffer 100 mM NaCl, 25 mM Tris/HCl, pH 8.0. The analytical standards were BSA (67 kDa), ovalbumin (43 kDa), ribonuclease (13.7 kDa), Aprotinin (6.5 kDa), vitamin B_12_ (1.3 kDa) of Superdex75 and Thyrogobulin (669 kDa), Ferritin (440 kDa), BSA (67 kDa), β-lactoglobulin (3 kDa), ribonuclease(13.7 kDa), Aprotinin(6.5 kDa), vitamin B_12_(1.3 kDa) of Superdex200. The control groups were only one-component protein in elution buffer (25 mM Tris/HCl pH 8.0, 100 mM NaCl). The experimental groups were carried out at three proteins of IspD, IspE, and IspF (0.2 mg), in a final volume of 250 μl; meanwhile, adding the 1 mM MgCl_2_, 1 mM MEP, and 1 mM CTP if the IspD was present in the reaction system. The reaction was incubated at 30°C for 30 min.

### Other methods

The cavity analysis was performed using CASTp server [37], surface calculations were performed by ArealMol from CCP4 programs suite [33].

### Accession numbers

The atomic co-ordinates and structure factors have been deposited in the Protein Data Bank with accession codes 5IWX (native) and 5IWY (CMP complex), respectively.

## Results

### Overall structure of *Bs*IspF

**Figure 2:**
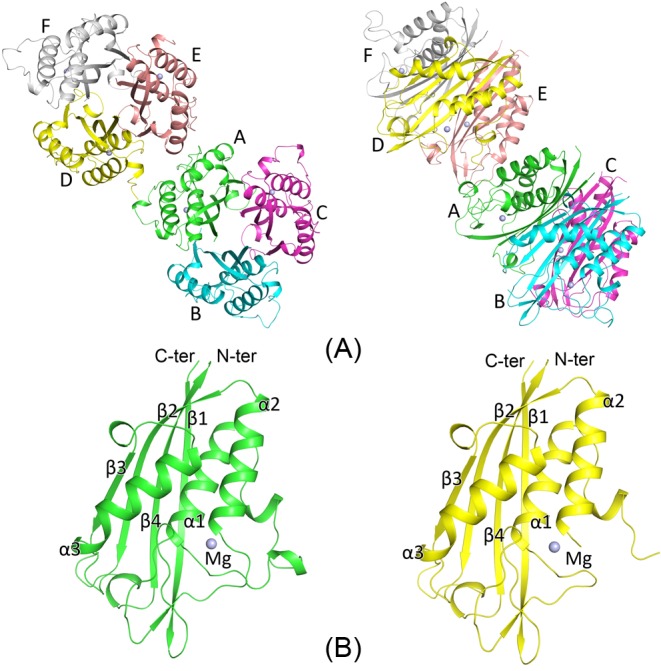
Overall structure of the *Bs*IspF trimer and monomer (**A**) Molecule packing in the asymmetric unit, with six monomers arranged into two trimers. (**B**) The monomer structure. Secondary structures and the magnesium ions are labeled.

The *Bs*IspF crystallizes in space group *P*2_1_, with unit-cell parameters *a* =56.96, *b* =89.30, *c* =84.71 Å, *β* =100.85°. The crystallographic asymmetric unit contains six monomers, which are arranged as two trimers of closely associated subunits. The monomer displays an α/β fold constructed with a four-strand β-sheet of β1, β2, β3, and β4, with β2 antiparallel to the others on one side, and a three-helix bundle of α1 to α3 on the other. In *Bs*IspF trimer, the four-strand β-sheet of each subunit lay roughly parallel to the threefold NCS axis, forming a compact bell-shaped trimer of approximately 45 × 60 Å in the axial and equatorial dimensions, respectively (Figure 2). Overall, the two trimers are extremely similar with a root mean square deviation (r.m.s.d) value of 0.20 Å over all Cα atoms. The largest Cα r.m.s.d differences (~1.5 Å) between the two trimers occur in two loop regions, the first one connects β1 to α1 with the residues 34–39, and the second one links α1 to α2, which comprises residues 62–72. Both loops are parts of the active pocket and involved in the binding of 2-C-methyl-d-erythritol (MEPP) moiety.

**Figure 3:**
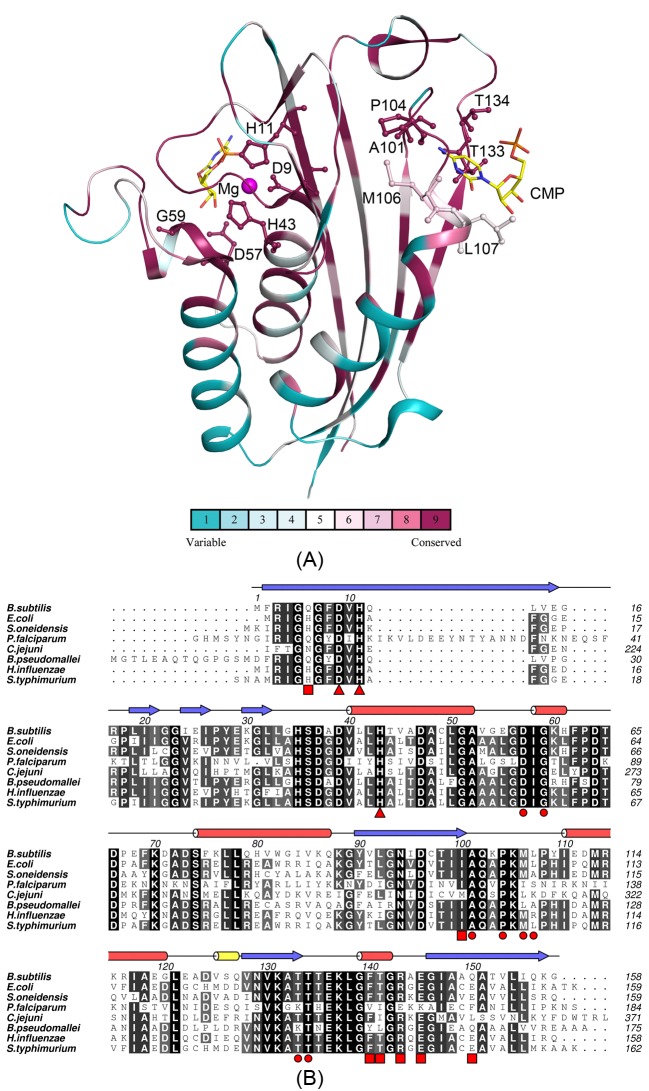
Sequence conservation of *Bs*IspF structrure (**A**) Sequence-conservation scores of *Bs*IspF monomer [50]. Residues that contribute to the interactions with the CMP are shown as sticks; the most variable residues are shown in blue and the most conserved residues are shown in dark purple. (**B**) Multiple sequence alignment IspF protein. Sequences of IspF from *B. subtilis, E. coli, S. oneidensis, P. falciparum, C. jejuni, B. pseudomallei, H. influenzae, S. typhimurium* (UniProt identifiers: Q06756, P62617, Q8EBR3, P62368, Q9PM68, Q3JRA0, P44815, Q8ZMF7, respectively) were aligned with MUSCLE [51] and edited by hand to match the structural similarity where appropriate by using ALINE [52]. Identical and similar residues were highlighted in black and gray, respectivey. The secondary structure elements base on the *Bs*IspF monomer, *α*-helix and *β*-strand are marked by red pillar and blue arrow, respectively. Three residues that co-ordinate the magnesium ions are marked with red triangles. Residues that bind CMP are marked with red circles. Residues that form the hydrophobic cavity are highlighted with red squares.

**Figure 4:**
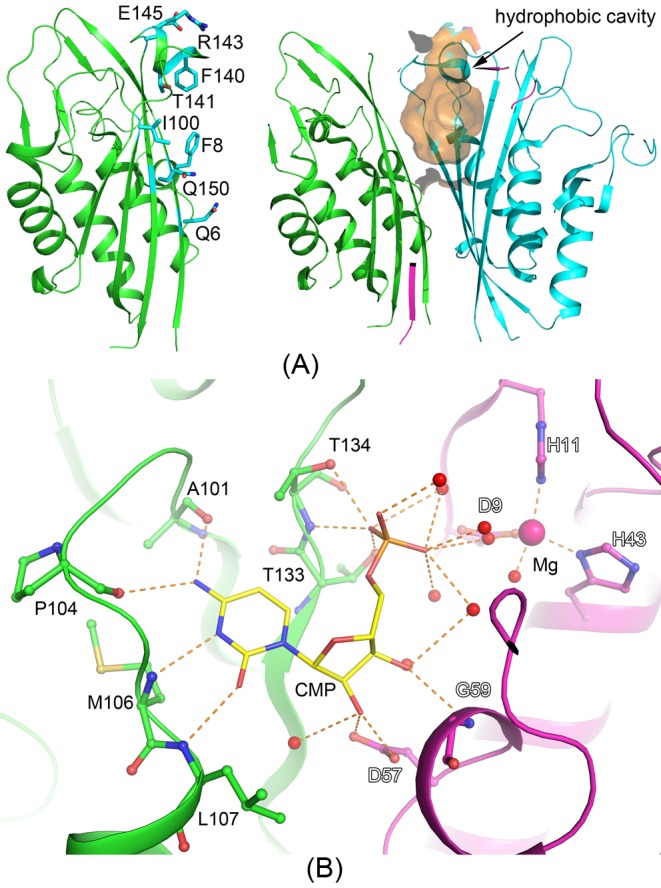
Representative of the hydrophobic cavity and the interactions between CMP and *Bs*IspF (**A**) Representative of the hydrophobic cavity in the *Bs*IspF trimer. Cartoon representations of *Bs*IspF subunits (left) and a section of the overall structure of *Bs*IspF trimer (right). The residues involved in the hydrophobic cavity formation are shown as stick model and labeled. The hydrophobic cavity is shown in surface model and labeled. (**B**) Representative of the interactions between CMP and *Bs*IspF. The CMP molecule is shown as stick model in yellow. The magnesium ion and the water molecules are shown as sphere. The relevant residues from only two subunits are shown as stick model, with C positions colored green and purple, respectively. Hydrogen and co-ordination bonds are shown as orange dotted lines.

The *Bs*IspF shares a sequence identity of ~62% with IspF from *E. coli* (*Ec*IspF), and overlay between the *Bs*IspF and *Ec*IspF (PDB: 1KNK) trimer give an r.m.s.d value of 0.86 Å, indicating a high structural similarity. The superimposition of structures of *Bs*IspF and *Ec*IspF are shown in Supplementary Figure S1. However, although the accessible surface area (ASA) of a *Bs*IspF monomer (7584 Å^2^) is nearly the same as ASA of *Ec*IspF monomer (7681Å^2^), the ASA of the *Bs*IspF trimer (17966 Å^2^), is larger than that of *Ec*IspF trimer (16293 Å^2^), which demonstrates a looser packing of the subunits of *Bs*IspF. Compared with the structure of *Ec*IspF, there is also a hydrophobic cavity along the three-fold symmetry axis of *Bs*IspF homotrimer (Figure 4A). Several aliphatic amino acids from three subunits line inside of the cavity (Phe^8^, Ile^100^, and Phe^140^). The bottom of the cavity is closed by the hydrogen bonds involving six glutamines (Gln^150^ and Gln^6^), and the cap of the cavity is narrowed to a 5-Å diameter hole by the residues Arg^143^ and Glu^145^ from three subunits. Sequence alignment reveals that all the residues mentioned above are highly conserved between *Ec*IspF and *Bs*IspF enzymes, except the residues Gln^150^ and Gln^6^ in *Bs*IspF (corresponding residues Glu^149^ and His^5^ in *Ec*IspF) (Figure 3B). The volume of the cavity of *Bs*IspF (1024Å^3^) is larger than that of *Ec*IspF (718Å^3^), which is consistent with the looser packing of the subunits in *Bs*IspF. In other reported ispF structures, such as *E. coli, S. oneidensis* and *C. jejuni* etc., electron density had been found inside the cavities, which was interpreted as isoprenoid species such as geranyl- or farnesyl-phrophosphate, with a possible feedback-regulatory role through a yet undetermined mechanism, which might be related to the trimer stability [15,19,38–42]. However, not all ispF enzymes have isoprenoid species binding in the cavity. For the structure of IspF from *Haemophilus influenzae* [43], the density inside the cavity was interpreted as a sulphate ion, for the structure of IspF from *Burkholderia cenocepacia* [44], a molecule of di(hydroxyethyl)ether, a likely decomposition product or impurity of PEG3350 used in crystallization, occupies the cavity, and for the structure of IspF from *Plasmodium falciparum*, only a hollow cavity is observed. In addition, such a cavity is absent from the structure of IspF from *Thermus thermophilus* [45], where the packing of subunits is tighter and likely more stable according to ultracentrifugation data. Besides, in the IspF structure from *Arabidopsis thaliana*, the cavity is unsuited for binding a diphosphate moiety, which suggests a different regulatory mechanism of IspF enzymes between bacteria and plants [46]. In *Bs*IspF, no ligand binding in the cavity is observed because no obviously continuous electron density is found here, and only a hollow cavity is presumed in both native and *Bs*IspF/CMP complex, but regarding the highly conserved residues involved in the cavity formation between *B. subtilis* and *E. coli* and the size or dimension of the cavity in *Bs*IspF, potential ligands of isoprenoid species could bind here. Till now, no accurate reports are proposed to elucidate the mechanism of the possible feedback-regulatory role by the hydrophobic cavity in the IspF enzymes, further investigations are required to clarify and that is what we will do.

### Active pockets

*Bs*IspF trimer have three active pockets which are formed by the interface between two adjacent subunits, and here the magnesium ions deriving from the magnesium formate dehydrate used in crystallization are co-ordinated by the side chains of two histidines and an aspartate (Asp^9^, His^11^, and His^43^). Generally, the IspF enzymes require the divalent cation to position the substrate and stabilize the charge in the transition state during the catalytic reaction. In other structures of IspF proteins, the manganese or zinc ions were commonly bonded in the active site [15,38], meanwhile the magnesium ions were also substituted in the IspF structure from *T. thermophilus* [45]. In both native and CMP bonded structures of *Bs*IspF, magnesium ions were refined in the active site, with the ingredient of magnesium ions used in the crystallization cocktail. Moreover, in our structures, the B-factors of magnesium ions were refined well in both native and complex structures. Structure-based sequence alignment reveals that residues around the active pocket are well conserved in all the IspF enzymes (Figure 3). And, the overlay of each chain between the native and *Bs*IspF/CMP complex reveal r.m.s.d values ranging from 0.12 to 0.24 Å. The active sites comprise a nucleotide binding pocket, a cation binding site, and two loops for the binding of the MEPP moiety. The nucleotide binding pocket and cation binding site are relatively rigid, with a main-chain B-factor of 37.1 Å^2^, which is basically consistent with the average B-factor of the main-chain protein atoms, 35.2 Å^2^. Moreover, structural comparisons show that only a small Cα r.m.s.d differences (~0.1 Å) occur in this region between *Bs*IspF and *Bs*IspF/CMP complex. However, the two loops binding for the MEPP moiety are rather flexible. The first loop (residues: 34–39) displays elevated main-chain B-factors of 45.1 Å^2^ compared with 42.2 Å^2^ in *Bs*IspF and *Bs*IspF/CMP complex, respectively. The electron density of the second loop (residues: 62–72) in *Bs*IspF and *Bs*IspF/CMP complex is of insufficient quality to allow this region to be modeled, indicating a high degree of flexibility. CMP acts as the leaving group to give the cyclic diphosphate reaction product MEcDP. In the *Bs*IspF/CMP complex, only four CMP moieties can be modeled in the six active pockets of the two trimers in the asymmetric unit with the occupancy higher than 0.7, one trimer fully loads CMP substrates, and the other captures only one. Although the two trimers in the asymmetric unit display extremely structural similarity (r.m.s.d: 0.20 Å), the B-factors of the active pocket in the two trimers are quite different, which may be the possible cause for this asymmetric binding (Supplementary Figure S2). The CMP moieties reveal similar interactions with the protein. The cytidine group of CMP interacts with the residues Ala^101^, Pro^104^, Met^106^, and Leu^107^ through main-chain atoms, and the ribose hydroxyl groups form hydrogen bonds with the carboxylate of Asp^57^ and the amide of Gly^59^ from the adjacent subunits. Residues Thr^133^ and Thr^134^ hydrogen bond to the α-phosphate of CMP, waters act as a bridge between the α-phosphate and the magnesium ion (Figure 4B). All these residues are highly conserved in the IspF protein (Figure 3).

We analyzed the ASA of active pockets in the native IspF structures which had been deposited in the PDB bank. The IspF enzymes from pathogens basically display a small ASA of its active pockets. The IspF from *Salmonella typhimurium* (PDB: 3GHZ) and *H. influenzae* [43] revealed minimum-size active pocket with an ASA of ~610 Å^2^, whereas the IspF from *Plasmodium falciparum* [44], *Burkholderia pseudomallei* [47], and bifunctional IspDF from *C. jejuni* [19] show a medium-size active pocket with an ASA ranging from ~900 to 950 Å^2^, the rest IspF enzymes show a large-size active pocket, like IspF from *S. oneidensis* [39], *E. coli* [38], *B. subtilis* etc. The diversity of the primary sequence in difference organisms may lead to the variation on the active pocket. Furthermore, we focussed our study on the two model organisms, *B. subtilis* and *E. coli*. The primary sequences of the active pockets are strictly conserved in the *Bs*IspF and *Ec*IspF, except the residue His^62^ in the *Bs*IspF (corresponding residue Leu60 in *E. coli*, which have an van der Waals interaction between the methyl group on MEcDP in the *Ec*IspF/MEcDP complex [38]). However, cavity analysis revealed that the ASA of active pocket in native *Bs*IspF is approximately 1020 Å^2^ compared with 1150 Å^2^ in native *Ec*IspF (PDB:1KNK), whereas the ASA of active pocket in *Bs*IspF/CMP complex is approximately 820 Å^2^ compared with 910 Å^2^ in *Ec*IspF/CMP complex (PDB:1H47) [38,41]. Although a looser packing of the subunits of *Bs*IspF, the active pockets of *Bs*IspF are tighter than that of *Ec*IspF in native or in complex with CMP.

### Protein associations

**Figure 5:**
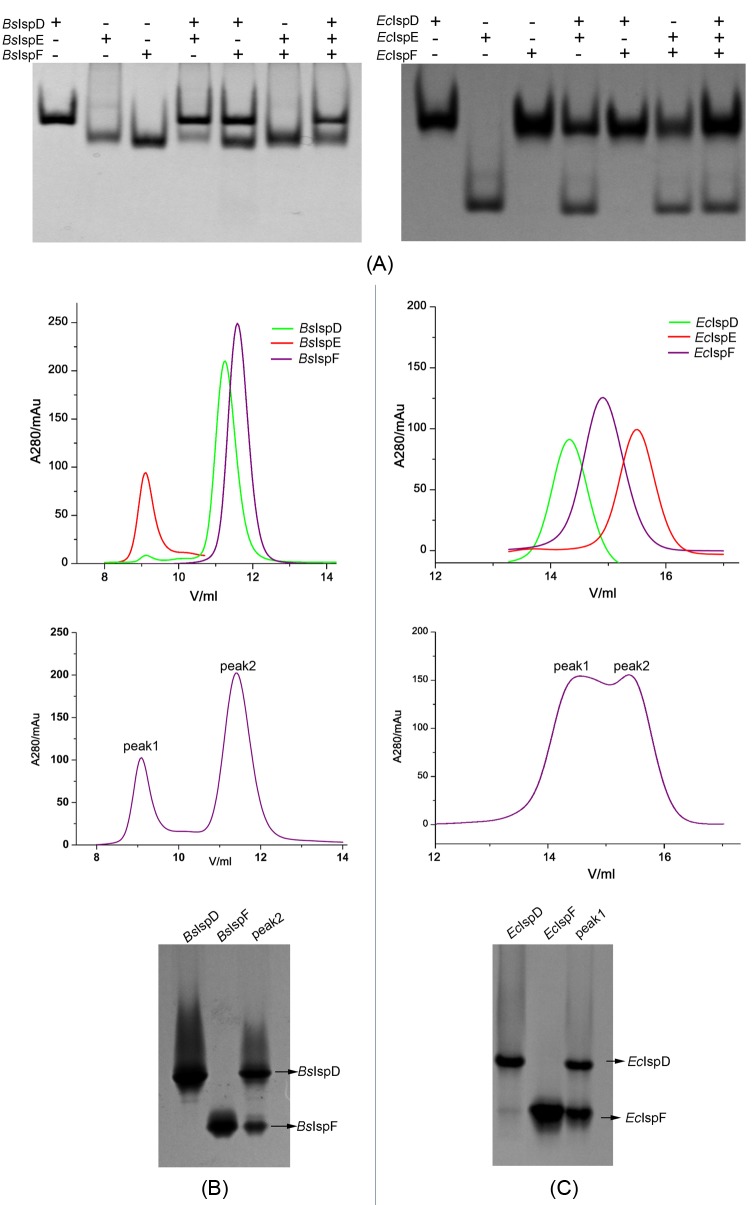
Analysis on the associations of IspD, IspE, and IspF (**A**) Three individual proteins (IspD, IspE, and IspF), dual mixture (IspD/IspE, IspD/IspF, and IspE/IspF) and triple mixture (IspD/IspE/IspF) from *B. subtilis* (left) and *E. coli* (right) are analyzed by native gel shift, respectively. (**B**) Three individual proteins (green, red, and purple corresponding to *Bs*IspD, *Bs*IspE, and *Bs*IspF, respectively) (upper), and triple mixture (*Bs*IspD/*Bs*IspE/*Bs*IspF) (middle) are analyzed by size exclusion chromatography, and the samples from the peak2 are re-analyzed by native gel shift (lower). (**C**) Three individual proteins (green, red, and purple corresponding to *Ec*IspD, *Ec*IspE, and *Ec*IspF, respectively) (upper) and triple mixture (*Ec*IspD/*Ec*IspE/*Ec*IspF) (middle) are analyzed by size exclusion chromatography, the samples from the peak1 are re-analyzed by native gel shift (lower).

Protein associations amongst IspD, IspE, and IspF enzymes are investigated by native gel shift. In *B. subtilis*, no stable complexes of *Bs*IspD/*Bs*IspE, *Bs*IspD/*Bs*IspF, and *Bs*IspD/*Bs*IspE/*Bs*IspF have been observed (Figure 5A). Otherwise, similar results are observed in *E. coli*, referring to *Ec*IspD/*Ec*IspE, *Ec*IspE/*Ec*IspF, and *Ec*IspD/*Ec*IspE/*Ec*IspF (Figure 5A). However, the dual mixture of *Bs*IspE and *Bs*IspF seems to merge into a single band. Similar results are observed in the dual mixture of *Ec*IspD and *Ec*IspF (Figure 5A). Therefore, the size exclusion chromatography method is performed to verify whether a stable complex could be formed.

*Bs*IspD, *Bs*IspE, and *Bs*IspF are dimer (51.6 kDa), monomer (57.7 kDa, a fusion protein of GST-*Bs*IspE) and trimer (51.3 kDa), respectively. As shown in Figure 5B, *Bs*IspD, *Bs*IspE, and *Bs*IspF are eluted at ~11.25, 9.11, and 11.58 ml by a Superdex75 column, respectively. However, the mixture of triple enzymes is eluted at wo peaks, ~9.1 and 11.4 ml. Compared with the profile of each single protein, the peak1 (~9.1 ml) exhibits a monomeric *Bs*IspE and peak2 (~11.4 ml) may represent mixture of a dimer *Bs*IspD, a trimer *Bs*IspF, or the complex of *Bs*IspD/*Bs*IspE. Therefore, no complex formation of *Bs*IspE/*Bs*IspF is detectable. Furthermore, the samples from peak2 are analyzed by native gel shift, but no *Bs*IspD/*Bs*IspF complex is observed. So in the *B. subtilis*, no dual complex or triple complex is formed between/amongst the IspD, IspE, and IspF *in vitro* (Figure 5B).

*Ec*IspD, *Ec*IspE, and *Ec*IspF are dimer (51.4 kDa), monomer (30.9 kDa ) and trimer (50.7 kDa), respectively. As shown in Figure 5C, *Ec*IspD, *Ec*IspE, and *Ec*IspF are eluted at ~14.3, 15.5, and 14.9 ml, respectively. The mixture of triple enzymes can be resolved by a Superdex200 column into two singlet peaks, ~14.6 and 15.4 ml. Compared with the profile of the single protein, the peak1 (~14.6 ml) may represent mixture of a dimer *Ec*IspD, a trimer *Ec*IspF or the complex between them, the peak2 (~15.4 ml) reveals the monomer *Ec*IspE. Samples from peak1 are re-analyzed by native gel shift, no *Ec*IspD/*Ec*IspF complex is observed. Therefore, in the *E. coli*, no dual complex or triple complex is formed between/amongst the IspD, IspE, and IspF *in vitro*, either (Figure 5C).

## Discussion

Nowadays, structural and biochemical studies of IspF and a number of ligands complexes from several species have been reported, which had produced an excellent understanding of substrate recognition and mechanism of catalysis by IspF enzyme [15,38–41,43–46,48]. In this study, crystal structures of *Bs*IspF in native and complexed with CMP have been determinated both at 1.99-Å resolutions. *Bs*IspF reveals a trimeric enzyme with three active pockets. Residues around the active pocket are well conserved. The parts of active pocket, nucleotide and cation binding sites are relatively rigid, whereas the other part of pocket that binds the methylerythritol-phosphate portion of the substrate is relatively flexible. These flexible loops may assist in the successive conformational change of the active pocket, and benefit the substrates binding, intermediate forming, and products releasing [28]. The *Bs*IspF trimer has high structural similarly with the *Ec*IspF, only differences are observed in the packing of the subunits and the solvent accessible surface of active pockets. Sequence alignment reveals that the primary sequences of active pockets in *B. subtilis* and *E. coli* are highly conserved (except one residue). It is likely that the active pockets in *Ec*IspF are more exposed to the solvent than in *Bs*IspF. These surface variations may mainly result from the packing in the interface between two adjacent subunits, and lead to the difference on the active pockets, such as the size and the shape, which may affect the binding of the substrates and relate to the efficiency of enzyme [49]. In our previous work, the enzyme IspD from *B. subtilis* presents a higher CTP hydrolytic activity and also a tighter active pocket than that in *E. coli* [28]. Perhaps, the specific property of active pocket in *Bs*IspF may relate to the catalytic efficiency of *Bs*IspF in MEP pathway.

Previous studies had focussed on the IspDF bifunctional enzyme that catalyzed the third and fifth steps in MEP pathway steps and proposed possible associations could be formed between bifunctional IspDF and monofunctional IspE or the individual enzymes IspD, IspE, and IspF [17,19], however, further studies rejected this hypothesis [20]. In our work, whether a multienzyme complex can be formed *in vitro* amongst the monofunctional enzymes IspD, IspE, and IspF from *B. subtilis* and *E. coli* are analyzed by the traditional methods, native gel shift and size exclusion chromatography methods. Initially, we conceived if a multienzyme could be stably formed amongst the IspD, IspE, and IspF proteins, using structural biology methods, such as crystallography or cryo-EM, to investigate the protein complex assembly was both attractive and challenging. However, in our study, no protein complex assembly amongst IspD, IspE, and IspF is observed in *E. coli* and *B. subtilis in vitro*, which shows the structural biology methods are unsuited for further analysis for the proteins complex assembly of individual IspD, IspE, and IspF. Furthermore, a comparison between the structures of the monofunctional protein, *Ec*IspD, *Ec*IspF, *Bs*IspD, *Bs*IspF, and the fused protein IspDF from *C. jejuni* indicate that there have been some differences amongst them. Structure of *Cj*IspDF reveals two distinct domains, corresponding to the cytidyltransferase IspD (N-terminal domain) and synthase IspF (C-terminal domain), respectively [19]. The two domains are connected by a linker loop comprising eight residues. The interface between the two domains in *Cj*IspDF is mainly hydrophobic, and consists of several aliphatic amino acids, such as leucines, isoleucines, phenylalanines etc. [19]. These hydrophobic interactions may have an advantage on the stabilization of the fused protein. However, the corresponding region of the individual *Ec*IspD (*Bs*IspD), *Ec*IspF (*Bs*IspF) is dominanted by polar amino acids, such as the glutamates and asparagines. These features may be unfavorable for the stable interaction between IspD and IspF. It must be pointed out that the native gel shift and size exclusion chromatography methods are limited tools for the analysis of proteins assembly *in vitro*, especially for the weak affinity by a complex formation. Perhaps, weak interactions may still be relevant in the crowded solutions found in the bacterial cytoplasm or the associations amongst IspD, IspE, and IspF might be transient in the bacterial cytoplasm, which is undetectable using the current methods *in vitro*. In fact, further investigations are required to clarify and the suitable methods, like NMR, CO-IP, SPR, etc. can be used in the weak interactions study, and that is what we will do in the next.

*B. subtilis* is a potential host cell for the production of isoprenoid. Several studies suggested that the enzymes of *B. subtilis* functioned more efficiently compared with that of other microorganisms [25–27,29]. In this work, the crystal structures of *Bs*IspF and *Bs*IspF/CMP complex have been determined. Structural comparisons reveal that the *Bs*IspF has high structural similarly with the *Ec*IspF, only differences are observed in the packing of the subunits and the solvent accessible surface of active pockets. In addition, the results show that the proteins complex assembly by IspD, IspE, and IspF enzymes in *B. subtilis* and *E. coli* are undetectable. The present study can supplement the high-resolution structures of the key enzyme and advance the understanding in the MEP pathway of *B. subtilis.* Probably, with advances in metabolic engineering, systems biology, and protein engineering, the *B. subtilis* strain can be optimized as an efficient Gram-positive host for the production of isoprenoids.

## Supporting information

**Figure S1:** The cartoon superimposition of *BsI*spF and *Ec*IspF subunit (a) or trimer (b) structures. The structures of *BsI*spF and *Ec*IspF are shown in green and gray, respectively.

**Figure S2:** Overall structure of the two *B*sIspF trimers in one asymemetric unit. Two view are shown, (a) top and (b) side view. The chain chains have been drawn in B-factors with the thicker tube represent the higher B-factors. The left trimer captures three CMP, Whereas the right trimer capture only one. The CMP is show in stick.

## Abbreviations

ASA: accessible surface area
*Bs*IspF: IspF protein of *B. subtilis*
CDPME: 4-diphosphocytidyl-2-C-methyl-d-erythritol
CDPME2P: 4-diphosphocytidyl-2-C-methyl-d-erythritol 2-phosphate
DMAPP: dimethylallyldiphosphate
*Ec*IspF: IspF from *E. coli*
GST: Glutathione-S-Transmerase
IPP: isopentenyldiphosphate
IspD: 2-C-methyl-d-erythritol-4-phosphatecytidyltransferase
IspE: CDPME kinase
IspF: 2-C-methyl-d-erythritol 2,4-cyclodiphosphate synthase
MEP: 2-C-methyl-d-erythritol-4-phosphate
MEcDP: 2-C-methyl-d-erythritol 2,4-cyclodiphosphate
MEPP: 2-C-methyl-d-erythritol
MVA: mevalonate
NCS: non-crystallographic symmetry
r.m.s.d: root mean square deviation
SSRF: Shanghai Synchrotron Radiation Facilities
TBE: Tris Boric acid EDTA
