# Supplementary material for: Crystal structure of IspF from Bacillus subtilis and absence of protein complex assembly amongst IspD/IspE/IspF enzymes in the MEP pathway
Source: Biosci Rep. 2018 Feb 21;38(1):BSR20171370. doi: 10.1042/BSR20171370 (PMC5821942; doi:10.1042/BSR20171370)
Supplement: Supplementary file 1 [file bsr20171370_Supp1.pdf]

Supplementary Materials

Crystal structure of IspF from *Bacillus subtilis* and absence of protein complex assembly among IspD/IspE/IspF enzymes in the MEP pathway

Zhongchuan Liu [1, 2], Yun Jin [1, 2, 4], Weifeng Liu [3], Yong Tao [3], Ganggang Wang [1, 2*]

[1] *Key Laboratory of Environmental and Applied Microbiology, Chengdu Institute of Biology, Chinese Academy of Sciences, Chengdu, 610041, China;*

[2] *Key Laboratory of Environmental Microbiology of Sichuan Province, Chengdu, 610041, China;*

[3] *Chinese Academy of Sciences Key Laboratory of Microbial Physiological and Metabolic Engineering, Institute of Microbiology, Chinese Academy of Sciences, Beijing 100101, People's Republic of China;*

[4] *University of Chinese Academy of Sciences, Beijing, 100049, China.*

*Corresponding author’s:

SEND CORRESPONDENCE TO:

Ganggang Wang

Key Laboratory of Environmental and Applied Microbiology, Chengdu Institute of Biology, Chinese Academy of Sciences, Chengdu, 610041, China

Tel: 86-28-82890828; Fax: 86-28-82890828; E-mail: wanggg@cib.ac.cn

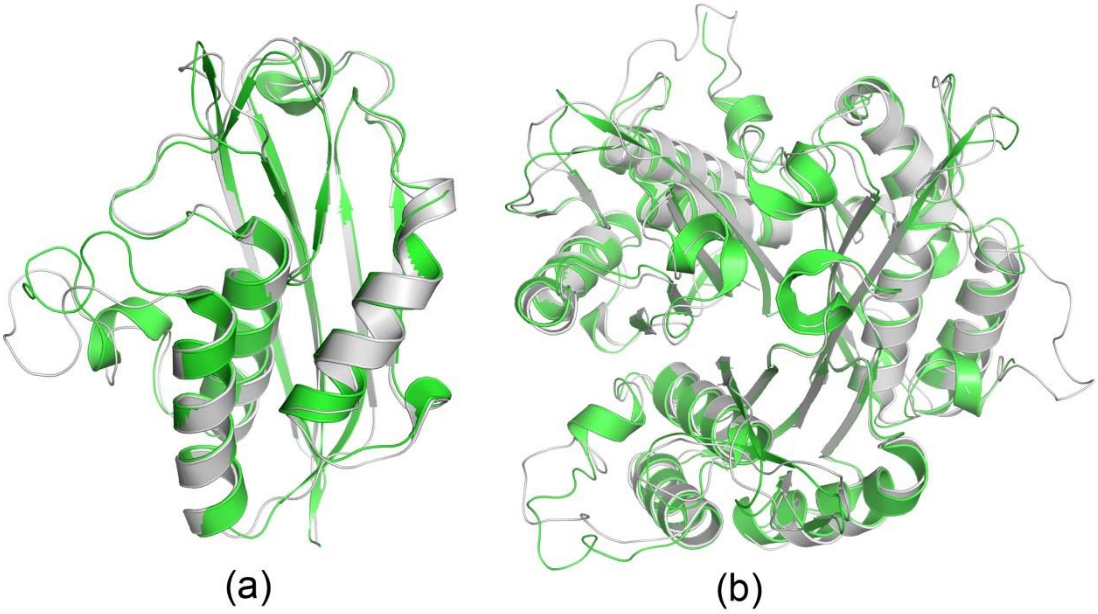

Figure S1: The cartoon superimposition of *BsI*spF and *Ec*IspF subunit (a) or trimer (b) structures. The structures of *Bs*IspF and *Ec*IspF are shown in green and gray, respectively.

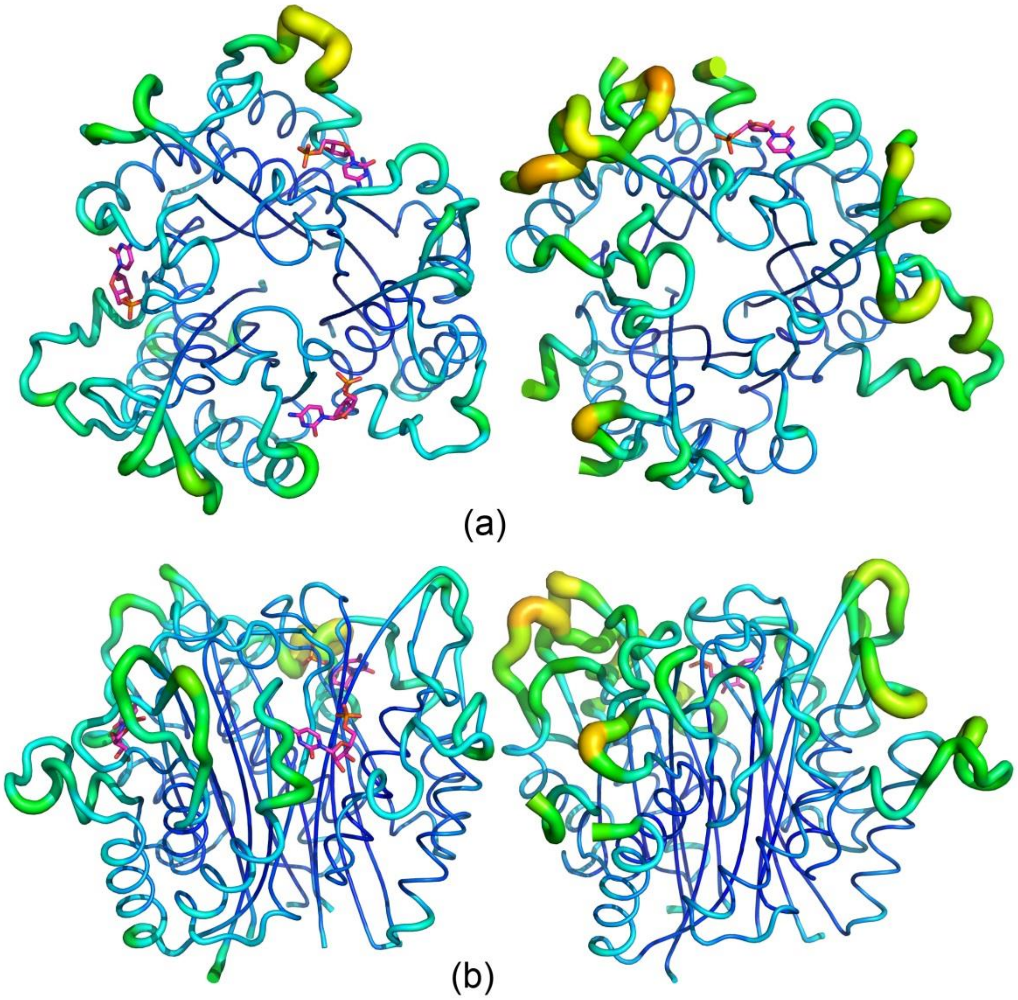

(a)

(b)

Figure S2: Overall structure of the two *B*sIspF trimers in one asymemetric unit. Two views are shown, (a) top and (b) side view. The chains have been drawn in B-factors with the thicker tube representing the higher B-factors. The left trimer captures three CMP, whereas the right trimer capture only one. The CMP is shown in stick.
